# Optimizing Traffic Engineering for Resilient Services in NFV-Based Connected Autonomous Vehicles

**DOI:** 10.3390/s21248446

**Published:** 2021-12-17

**Authors:** Tuan-Minh Pham, Thi-Minh Nguyen

**Affiliations:** 1 Faculty of Computer Science, Phenikaa University, Hanoi 12116, Vietnam; 2 A&A Green Phoenix Group JSC, Phenikaa Research and Technology Institute (PRATI), Hanoi 11313, Vietnam; 3 Department of Technology, Dong Nai Technology University, Bien Hoa City 760000, Vietnam; nguyenthiminh02@dntu.edu.vn

**Keywords:** NFV, VCAV, resilient service, optimization, reinforcement learning, connected autonomous vehicles

## Abstract

The massive amount of data generated daily by various sensors equipped with connected autonomous vehicles (CAVs) can lead to a significant performance issue of data processing and transfer. Network Function Virtualization (NFV) is a promising approach to improving the performance of a CAV system. In an NFV framework, Virtual Network Function (VNF) instances can be placed in edge and cloud servers and connected together to enable a flexible CAV service with low latency. However, protecting a service function chain composed of several VNFs from a failure is challenging in an NFV-based CAV system (VCAV). We propose an integer linear programming (ILP) model and two approximation algorithms for resilient services to minimize the service disruption cost in a VCAV system when a failure occurs. The ILP model, referred to as TERO, allows us to obtain the optimal solution for traffic engineering, including the VNF placement and routing for resilient services with regard to dynamic routing. Our proposed algorithms based on heuristics (i.e., TERH) and reinforcement learning (i.e., TERA) provide an approximation solution for resilient services in a large-scale VCAV system. Evaluation results with real datasets and generated network topologies show that TERH and TERA can provide a solution close to the optimal result. It also suggests that TERA should be used in a highly dynamic VCAV system.

## 1. Introduction

Recently, emerging Internet of Things (IoT) applications, such as connected autonomous vehicles (CAV), smart home, mobile augmented reality, smart agriculture, became increasingly popular [1]. A CAV system relies upon computer vision using a series of video cameras, radars, and Light Detection and Ranging (LIDAR) that allow the car to perceive the world around it. The system processes a massive amount of data collected from sensors to provide its application services composed of several application functions, including video capturing, sensor fusion, object tracking, localization, path planning, and control components. For example, a video camera on an autonomous car could generate hundreds of gigabytes in an hour of driving for a 720p video. A critical issue for a CAV system is how to transfer and process the massive amount of data generated daily in a timely fashion.

Network Function Virtualization (NFV) has been raised as a promising approach to tackling this issue [2]. A virtualized network function (VNF), including a traditional network function and general computation task, can be deployed as an instantiable software component running in a commercial off-the-shelf server. A VNF instance can be placed in edge devices to enable application services with low latency. Several VNFs on different edge and cloud devices can be connected as a service function chain (SFC) to enable real-time and flexible services. While an NFV-based CAV system, referred to as VCAV, is able to provide a flexible CAV service with low latency, it is challenging to protect a service from any system failure.

The issue of resilient services has been discussed in a specification published by The European Telecommunications Standards Institute (ETSI) [3]. The main challenge of providing a resilient service in a VCAV system is to optimize the placement and routing of VNFs in response to a failure in an NFV infrastructure (NFVI). Previous researches have considered various techniques to address several aspects of the resilient service problem [4,5,6,7,8,9]. However, all previous approaches could not be applied to a VCAV system due to the high dynamics of VCAV data traffic. In addition, most previous work assumes a fixed mapping of routing paths onto NFVI and implicit paths connected between VNFs in an SFC, which is not practical. Our work aims to optimize traffic engineering, including the VNF placement and routing for resilient services in a VCAV system, to minimize the service disruption cost, considering the dynamics of routing paths and service function chaining.

The main contributions of this paper are three-fold:

- We proposed an integer linear programming (ILP) model for the resilient service problem, referred to as TERO. The TERO model provides the optimal VNF placement and routing for a set of service demands when a failure occurs in a VCAV system.
- We developed a heuristic algorithm (i.e., TERH) and Reinforcement Learning (RL) based algorithm (i.e., TERA) to find an approximation solution for the resilient service problem in an extensive network. The approximation solution provided by TERH and TERA is close to the optimal solution. In comparison with TERH, TERA can achieve a similar cost with significantly reduced time in a dynamic failure scenario.
- We validate our proposed models and algorithms in real datasets and generated network topologies. The evaluation results suggest that TERA should be used to minimize the service disruption cost of a VCAV system concerning the high dynamics of data traffic.

The rest of the paper is organized as follows. Section 2 reviews some related works. Section 3 describes the system and states the optimization problem of traffic engineering for resilient services in a VCAV system. Section 4 presents the TERO model that provides the optimal traffic engineering for resilient services, including VNF placement and routing when a failure occurs in a VCAV system. Section 5 describes the TERH and TERA algorithms based on heuristic and reinforcement learning to find an approximation solution for the resilient service problem. We present the evaluation of our proposed model and algorithms in In Section 6. Finally, the conclusion is presented in Section 7.

## 2. Related Work

Network Function Virtualization (NFV) has been raised as a potential approach to a flexible and efficient solution for processing a massive volume of data in an IoT system. For the evolution of an IoT system with NFV, we refer the readers to [10]. The reliability of services on the Internet and in an IoT system is a crucial problem that has been widely studied (e.g., [11,12,13,14,15,16]). However, existing solutions are not suitable for an NFV-based IoT system, where functional modules can be deployed in different data centers and connected together to create a flexible service.

The challenge of developing a solution for resilient services in an NFV-based IoT system is to find the optimal resource allocation for data routing and processing when a failure occurs in a distributed system. So far, a few studies have considered the design of a resilient NFV-based IoT system [17,18,19,20]. Huang et al. devised a proactive fail-over mechanism based on failure prediction to enhance the resilience of NFV services deployed in a distributed edge network [17]. Ergenc et al. analyzed the complexity and boundaries of the problem as well as developed heuristics to increase the fault tolerance of an IoT network when there are some node and link failures [18]. Bakhshi et al. proposed a mathematical model for an SDN-based fault-tolerant architecture in an IoT environment [19]. Sanabria et al. used machine learning techniques to provide prediction and alert capabilities for telemedicine applications [20]. They proposed a hybrid Edge/Cloud architecture training of the deep learning prediction model. Several optimization models have been proposed for resilient services in the Mobile Edge Computing [21]. However, these solutions lack considering service function chaining that is a key feature of NFV. In addition, the dynamic routing path has not been tackled while it is an essential feature of a CAV system. Some studies have discussed an efficient design for data processing and routing in a VCAV system (e.g., [22,23,24]) However, a solution to the traffic engineering problem for resilient services in a VCAV system has not been provided.

This paper offers a new ILP formulation for a traffic engineering solution, including the VNF placement and routing when node or link failures occur in a VCAV system. Moreover, we take into account the dynamic routing path at the request time. We also propose two algorithms based on heuristics and reinforcement learning to provide an approximation solution in a large-scale VCAV system.

## 3. System Description

**Figure 1:**
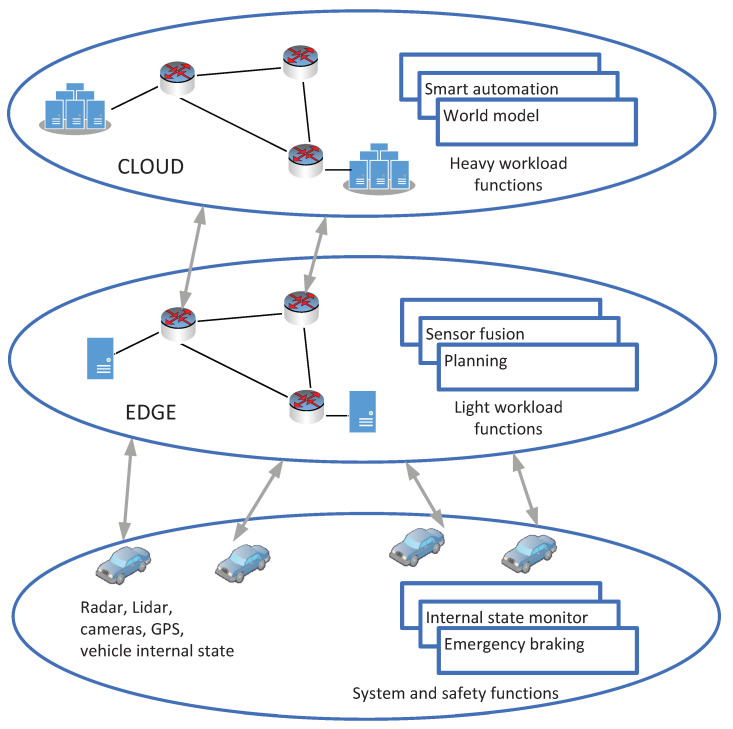
Functional components in an NFV-based connected autonomous vehicle.

In a VCAV system, system and safety functions are deployed locally in an autonomous vehicle. Light workload functions such as planning and sensor fusion can be run in edge nodes. Some functions that require a heavy computational task and process a massive volume of data collected from many cars can be implemented in the cloud layer (Figure 1). These functions can be connected in an order list to create an application service. An example of an SFC in a VCAV system is sensor fusion, world model, behavior generation, planning, and vehicle control. A VCAV system allocates its resource in the edge and cloud layers for a set of service demands required by vehicles. When a failure occurs, system resources are rapidly reallocated to maintain application services supplied to vehicles.

A CAV system based on NFV includes three main elements: the NFV infrastructure (NFVI), the VNFs, and the management and orchestration of NFVs (MANO). NFVI consists of the shared and virtualized resources of physical networking, computing, and storage. A VNF can be any functional module of a VCAV system, e.g., sensor fusion, world model, and planning. The MANO element handles all automatic processes for loading and managing VNFs. A traffic engineering solution for resilient services can be incorporated into the NFV architecture as a part of MANO.

We represent a VCAV system as a directed graph G=(V,E) where *V* and *E* denote the set of physical nodes and links. We define $r_{v}^{n}$ to be node *v*’s resource capacity, and $r_{e}^{l}$ to be link *e*’s bandwidth capacity. The beginning node and ending node of link *e* are denoted by $i_{e}$ and $j_{e}$. The node’s processing resource considered in this work is the number of CPU cores. We can use similar formulas of the processing resource in the model to include additional types of resources (e.g., memory, storage). We represent different network topologies by setting the parameters of links and nodes in the model. We denote by *F* the set of VNF types. $\eta_{u}$ is the number of cores required by VNF type u∈F to process a traffic volume. The routing delay $\beta_{v}$ is the time duration needed by node *v* to route an amount of traffic. The processing delay $\mu_{vu}$ is the time duration required to provide VNF type *u* at node *v*. We denote by $w=\left(w_{e}\right)$ the weight vector of NFVI where $w_{e}$ is an integer number representing link *e*’s weight. We define $\lambda_{v}^{n}$ to be the failure state of node *v*, and $\lambda_{e}^{l}$ to be the failure state of link *e*. $\lambda_{v}^{n}=0$ if node *v* fails, otherwise $\lambda_{v}^{n}=1$. $\lambda_{e}^{l}=0$ if link *e* fails, otherwise $\lambda_{e}^{l}=1$. A link failure can be caused by hardware problems, software issues (e.g., too many connections, configuration changes, denial of service attacks), or the mobility of vehicles.

We define $\Omega =\left\{S_{i}\right\}$ to be all system-supported SFC. An SFC is denoted by $S_{i}=\left(u_{i1},\ldots ,u_{ij},\ldots ,u_{in}\right)$ where $u_{ij}$ is the *j*th VNF of SFC $S_{i}$. The service demand set is denoted by $\Gamma =\left\{d\right\}$. The parameter set of service demand d∈Γ includes arrival node $s_{d}$, departure node $t_{d}$, SFC $S_{d}\in \Omega$, SFC delay $\alpha_{d}$, and bandwidth volume $b_{d}$. An arrival node is an NFV node that provides an entry of a service demand into a VCAV system. A departure node is an NFV node at which the demand traffic leaves a VCAV system. A middle node is an NFV node between an arrival node and a departure node on an SFC path realizing a service demand. An NFV node either provides a VNF instance or routes traffic of a service demand.

When a failure happens, a VCAV system needs to modify some paths of service demands and VNF placement on these paths to meet the requirement of service demands and avoid an overload of some nodes. The process is referred to as the traffic engineering problem for resilient services. Optimizing VNF placement and routing could significantly impact the cost efficiency and performance of a VCAV system. The problem is stated as follows:

**Problem** **1.** (Traffic Engineering for Resilient Services (TER)). *Given a VCAV system G, find a traffic engineering solution for fulfilling a service demand set* Γ, *in order to minimize the system interruption when failures occur under constraints on service functions chaining and the restriction rule of routing reallocation.*

## 4. Optimization Model for Resilient Services

We propose an optimization model based on ILP to find the optimal result of the TER problem. The model is referred to as TERO. The main variables of TERO are as follows:

- $x_{2}=\left(x_{2ed}\right)$ is the routing solution satisfying the service demand set when a failure occurs in a VCAV system. If demand *d* uses link *e*, $x_{2ed}=1$, otherwise, $x_{2ed}=0$.
- $y_{2}=\left(y_{2vdi}\right)$ is the VNF placement solution in the failure state. If node *v* provides the *i*th VNF of demand *d*, $y_{2vdi}=1$, otherwise, $y_{2vdi}=0$.

We summarize the main mathematical notations of TERO in Table 1.

### 4.1. Service Function Chaining Routing

The four conditions of service function chaining routing in a VCAV system are as follows: the flow balance, function provision, function chain, and delay constraints. The flow balance condition guarantees to conserve the flow traffic of a service demand along its path. The function provision condition assures that the VCAV system provides all VNFs of a service demand. The function chain condition guarantees that all VNFs of a service demand are connected in sequence. The delay constraint assures the fulfillment of the end-to-end delay of an SFC.

We define $l_{v_{1}v_{2}}$ to be the length of the path from node $v_{1}$ to node $v_{2}$. Let θ be a large number. The balance condition is as follows:

(1) $$\begin{array}{c} \sum_{\left\{e:i_{e}=v\right\}}x_{2ed}-\sum_{\left\{e:j_{e}=v\right\}}x_{2ed}=0,\;\forall d,\forall v,v\neq s_{d},v\neq t_{d}, \end{array}$$

(2) $$\begin{array}{c} \sum_{\left\{e:i_{e}=s_{d}\right\}}x_{2ed}=1,\;\forall d, \end{array}$$

(3) $$\begin{array}{c} \sum_{\left\{e:j_{e}=t_{d}\right\}}x_{2ed}=1,\;\forall d, \end{array}$$

(4) $$\begin{array}{c} \left(x_{2ed}-1\right)\theta ⩽l_{i_{e}t_{d}}-w_{e}-l_{j_{e}t_{d}}⩽\left(1-x_{2ed}\right)\theta ,\;\forall d,\forall e. \end{array}$$

Equation (1) guarantees that there is one entering flow and one leaving flow at a middle node on the path of a service demand. Equation (2) assures that there is one leaving flow at the departure node of a service demand. Equation (3) assures that there is one entering flow at the arrival node of a service demand. Equation (4) guarantees that there is no cycles in a service demand path.

The function provision condition is as follows:

(5) $$\begin{array}{c} \sum_{v}y_{2vdi}=1,\;\forall d,\forall i, \end{array}$$

(6) $$\begin{array}{c} y_{2vdi}⩽\sum_{\left\{e:i_{e}=v\;\mathrm{or}\;j_{e}=v\right\}}x_{2ed},\;\forall v,\forall d,\forall i. \end{array}$$

Equation (5) assures that the VCAV system provides all VNFs required by a service demand. Equation (6) ensures that the VCAV system only selects a node on the path of demand *d* to allocate a VNF for the demand.

To represent the function chain condition, we add two additional binary variables $y_{2vdi}^{\sigma }$ and ${\bar{y}}_{2edi}^{\sigma }$. If a node between $s_{d}$ and *v* on the path realizing demand *d* provides the *i*th VNF of demand *d* (i.e., $u_{di}$), $y_{2vdi}^{\sigma }=1$, otherwise $y_{2vdi}^{\sigma }=0$. If link *e* is on the path realizing demand *d*, and a node between $s_{d}$ and $i_{e}$ on the path provides $u_{di}$, ${\bar{y}}_{2edi}^{\sigma }=1$, otherwise ${\bar{y}}_{2edi}^{\sigma }=0$. The constraint is as follows:

(7) $$\begin{array}{c} y_{2vdi}⩽y_{2vd\left(i-1\right)}^{\sigma },\;\forall e,\forall d,\forall i⩾1, \end{array}$$

(8) $$\begin{array}{c} y_{2vdi}^{\sigma }=y_{2vdi}+\sum_{\left\{e:j_{e}=v\right\}}{\bar{y}}_{2edi}^{\sigma },\;\forall e,\forall d,\forall i, \end{array}$$

(9) $$\begin{array}{c} x_{2ed}+y_{2i_{e}di}^{\sigma }-1⩽{\bar{y}}_{2edi}^{\sigma }⩽x_{2ed},\;\forall v,\forall d,\forall i, \end{array}$$

(10) $$\begin{array}{c} {\bar{y}}_{2edi}^{\sigma }⩽y_{2i_{e}di}^{\sigma },\;\forall e,\forall d,\forall i. \end{array}$$

Equation (7) guarantees that node *v* supplies demand *d* with $u_{di}$ if and only if $u_{d(i-1)}$ is fulfilled by either node *v* or its preceding node that belongs to the demand *d*’s path. Equation (8) guarantees that $y_{2vdi}^{\sigma }=1$ if and only if $u_{di}$ is delivered by a node between $s_{d}$ and *v* and the node belongs to the path realizing demand *d*. Note that we have the sum of ${\bar{y}}_{2edi}^{\sigma }$ on the right-hand side of Equation (8) because there might be several incoming links of node *v*. Equations (9) and (10) assures that ${\bar{y}}_{2edi}^{\sigma }=1$ if and only if link *e* belongs to the path realizing demand *d*, and VNF $u_{di}$ is deployed at either $i_{e}$ or its preceding node that belongs to the demand *d*’s path.

The SFC delay represents the sum of the routing delay and VNF processing delay at every node that belongs to the demand path. We express the condition as follows:

(11) $$\begin{array}{c} \sum_{e}\beta_{i_{e}}x_{2ed}b_{d}+\sum_{v}\mu_{vu_{di}}\sum_{i}y_{2vdi}⩽\alpha_{d},\;\forall d. \end{array}$$

### 4.2. Restriction Rule in Flow Reallocation

First, the demand traffic cannot routed through a failed node or link. The condition is as follows:

(12) $$\begin{array}{c} \sum_{d}b_{d}x_{2ed}⩽r_{e}^{l}\lambda_{e}^{l},\;\forall e, \end{array}$$

(13) $$\begin{array}{c} \sum_{v,d,i}b_{d}y_{2vdi}\eta_{u_{di}}⩽r_{v}^{n}\lambda_{v}^{n},\;\forall v. \end{array}$$

Equation (12) guarantees that the total traffic of all demands passing through a link cannot surpass its bandwidth capacity. Equation (13) guarantees that the number of cores that a node allocates to the VNFs of all demands cannot surpass the node capacity. Note that when a node and link fail, the system loses all capacity of the node and link.

Second, the resource allocation for a service demand without failures on its paths should not be changed. We introduce three binary variables $\lambda_{e}^{\prime }$, $\varphi_{ed}$ and $\varphi_{d}^{\sigma }$. $\lambda_{e}^{\prime }=1$ if and only if a failure occurs on link *e*, at node $i_{e}$, or at node $j_{e}$. $\varphi_{ed}=0$ if and only if $\lambda_{e}^{\prime }=1$ and link *e* is on the path realizing demand *d*. $\varphi_{d}^{\sigma }=0$ if and only if there exists a link or node failure on the path used by demand *d*. Let $x_{1}=\left(x_{1ed}\right)$ be the current routing solution. If demand *d* uses link *e*, $x_{1ed}=1$, otherwise, $x_{1ed}=0$. The condition is given by:

(14) $$\begin{array}{c} \lambda_{e}^{l}⩽{\lambda^{\prime }}_{e},\;\forall e, \end{array}$$

(15) $$\begin{array}{c} \lambda_{i_{e}}^{n}⩽{\lambda^{\prime }}_{e},\;\forall e, \end{array}$$

(16) $$\begin{array}{c} \lambda_{j_{e}}^{n}⩽{\lambda^{\prime }}_{e},\;\forall e, \end{array}$$

(17) $$\begin{array}{c} {\lambda^{\prime }}_{e}⩽\lambda_{e}^{l}+\lambda_{i_{e}}^{n}+\lambda_{j_{e}}^{n},\;\forall e, \end{array}$$

(18) $$\begin{array}{c} \varphi_{d}^{\sigma }x_{1ed}⩽x_{2ed},\;\forall d,\forall e, \end{array}$$

(19) $$\begin{array}{c} \varphi_{d}^{\sigma }⩽\varphi_{ed},\;\forall d,\forall e, \end{array}$$

(20) $$\begin{array}{c} 1-{\lambda^{\prime }}_{e}⩽\varphi_{ed},\;\forall d,\forall e, \end{array}$$

(21) $$\begin{array}{c} 1-x_{1ed}⩽\varphi_{ed}⩽2-x_{1ed}-{\lambda^{\prime }}_{e},\;\forall d,\forall e. \end{array}$$

Equations (14)–(17) guarantee that $\lambda_{e}^{\prime }=1$ if and only if we have either $\lambda_{e}^{l}=1$, $\lambda_{i_{e}}^{n}=1$, or $\lambda_{j_{e}}^{n}=1$, and $\lambda_{e}^{\prime }=0$ if and only if we have $\lambda_{e}^{l}=0$, $\lambda_{i_{e}}^{n}=0$, and $\lambda_{j_{e}}^{n}=0$. Equation (18) guarantees that the routing solution for demand *d* does not change if there is no failures on its path. Equation (19) ensures that $\varphi_{d}^{\sigma }=0$ if and only if $\varphi_{ed}=0$ for one of links along the path used by demand *d*. Equations (20) and (21) ensure that $\varphi_{ed}=0$ if and only if $\lambda_{e}^{\prime }=1$ and $x_{1ed}=1$.

### 4.3. Objective Function

Our objective is to minimize the service disruption cost. The service disruption cost of a service demand is the cost of moving its VNF state and data to a new node. It is in proportion to the time required to provide all services normally. Its unit of measurement is a derived unit of time. We denote by $\gamma_{vv^{\prime }u_{di}}$ the cost when moving *i*th VNF of demand *d* from *v* to $v^{\prime }$. Let $\rho_{vv^{\prime }}$ be the cost of the minimum-weight path from *v* to $v^{\prime }$. $\kappa_{u}$ is the size of the state and data of a VNF type *u*.

The service disruption cost of a VNF instance is given by:

(22) $$\begin{array}{c} \gamma_{vv^{\prime }u_{di}}=\rho_{vv^{\prime }}\kappa_{u_{di}}. \end{array}$$

We add an additional variable $z_{vv^{\prime }di}$ to compute the service disruption cost in a VCAV system when a failure occurs. In a failure state, if the *i*th VNF of SFC $S_{d}$ is moved from node *v* to node $v^{\prime }$, $z_{vv^{\prime }di}=1$, otherwise, $z_{vv^{\prime }di}=0$. Let $y_{1}=\left(y_{1vdi}\right)$ be the current VNF placement solution. If node *v* provides the *i*th VNF of SFC $S_{d}$, $y_{1vdi}=1$, otherwise, $y_{1vdi}=0$. The constraints on the value of $z_{vv^{\prime }di}$ are given by:

(23) $$\begin{array}{c} z_{vv^{\prime }di}⩽y_{1vdi},\;\forall v,\forall v^{\prime },\forall d,\forall i, \end{array}$$

(24) $$\begin{array}{c} z_{vv^{\prime }di}⩽y_{2v^{\prime }di},\;\forall v,\forall v^{\prime },\forall d,\forall i, \end{array}$$

(25) $$\begin{array}{c} y_{1vdi}+y_{2v^{\prime }di}-1⩽z_{vv^{\prime }di},\;\forall v,\forall v^{\prime },\forall d,\forall i. \end{array}$$

Equations (23)–(25) guarantee that $v^{\prime }$, $z_{vv^{\prime }di}=1$ if and only if we have $y_{1vdi}=1$ and $y_{2v^{\prime }di}=1$, otherwise, $z_{vv^{\prime }di}=0$.

The service disruption cost of a resource allocation solution when a failure happens is given by:

(26) $$\begin{array}{c} U=\sum_{v,v^{\prime },d,i}z_{vv^{\prime }di}\gamma_{vv^{\prime }u_{di}}. \end{array}$$

### 4.4. ILP Model for Resilient Services

The TER problem is to find a traffic engineering solution for minimizing a cost function of service disruption when a failure occurs in a VCAV system. The TERO model provides the optimal VNF routing and placement for the TER problem in a failure state. The formulation of the TERO model includes the objective function given by Equation (26) and the constraints given by Equations (1)–(25).

## 5. Approximation Algorithms

In the previous section, we proposed the TERO model to obtain the optimal solution for traffic engineering in a VCAV system when a failure occurs. An ILP solver is not able to handle a scenario with hundreds of nodes and thousands of demands since the number of variables in TERO comes to billions in such a large scenario. Hence, we propose two algorithms based on a heuristic approach and reinforcement learning to find an approximation solution for the TER problem in a large-scale VCAV system. The two algorithms use the similar input parameters of the TER problem, which are presented in Table 1.

### 5.1. Heuristic Algorithm

We propose a heuristic algorithm, namely TERH, based on the Simulated Annealing (SA). In TERH, we develop the structure of the resource allocation solution and the function of neighborhood selection for the TER problem. SA is a heuristic technique that finds the optimum for a global optimization problem [25]. The search method accepts a worse scenario with a certain probability of overcoming a local optimum.

We represent a resource allocation solution for a service demand set in a VCAV system as a list of tuples, which is denoted by $O_{m}=\left(\left(d,i,v\right):d\in D,i\in S_{d},v\in V\right)$. The solution shows that node *v* provides the *i*th VNF of demand *d*. The details of the TERH algorithm are presented in Algorithm 1.
**Algorithm 1** Simulated Annealing-based approximation algorithm for TER1:**function** TERH(*G*, Γ, $y_{1}$)2:    Initialize *T*, $T_{0}$, $T_{n}$, ϕ, τ3:    Find an initial solution $O_{m}$4:    $O_{m}^{*}\leftarrow O_{m}$5:    Compute $y_{2}^{*}$ from $O_{m}^{*}$6:    **while** $T\geq T_{n}$ **do**7:        **for** n←1 to ϕ **do**8:           **repeat**9:               $\left(d,i,v\right)\leftarrow$ a random tuple in $O_{m}$10:               $v^{\prime }\leftarrow$ a random node in *V*11:               $O_{m}^{{}^{\prime }}\leftarrow R_{\mathrm{EPLACE}}\left(d,i,v,v^{\prime },O_{m}\right)$12:               Compute $x_{2}^{\prime }$ and constraints in a failure scenario13:           **until** $O_{m}^{\prime }$ is feasible14:           Compute $y_{2}$ from $O_{m}$15:           Compute $y_{2}^{\prime }$ from $O_{m}^{\prime }$16:           **if** $U(y_{2}^{\prime },y_{1})<U\left(y_{2},y_{1}\right)$ **then**17:               $O_{m}\leftarrow O_{m}^{\prime }$18:               **if** $U\left(y_{2}^{\prime },y_{1}\right)<U\left(y_{2}^{*},y_{1}\right)$ **then**19:                   $O_{m}^{*}\leftarrow O_{m}^{\prime }$20:                   $x_{2}^{*}\leftarrow x_{2}^{\prime }$21:                   $y_{2}^{*}\leftarrow y_{2}^{\prime }$22:               **end if**23:           **else**24:               $\Delta \leftarrow U\left(y_{2}^{\prime },y_{1}\right)-U\left(y_{2},y_{1}\right)$25:               ε← a random number between 0 and 126:               **if** exp(−Δ/T)>ε **then**27:                   $O_{m}\leftarrow O_{m}^{\prime }$28:               **end if**29:           **end if**30:        **end for**31:        T←C(T)32:    **end while**33:    **return** $y_{2}^{*}$, $x_{2}^{*}$34:**end function**

The algorithm contains two main loops. The outer loop is controlled by the temperature parameter *T*, the start temperature parameter $T_{0}$, the stop temperature parameter $T_{n}$, and the cooling function C(T). For each *T*, the algorithm runs an inner loop that uses a neighborhood function to move from the current solution to another. *T* decreases by C(T) after one iteration of the outer loop. The algorithm completes its solution search when *T* is smaller than $T_{n}$.

We define ϕ to be the number of iterations of the inner loop. Let $O_{m}$ be an initial solution. We use the most common cooling function C(T)=τT, for some parameter τ from interval (0, 1). The initial temperature is the maximal cost difference between any two neighbor solutions. The end temperature typically is close to zero.

In the neighborhood selection (i.e., line 8–12), we define the Replace $\left(d,i,v,v^{\prime },O_{m}\right)$ operator that substitutes node $v^{\prime }$ for node *v*. We use the Replace operator for a random tuple $\left(d,i,v\right)\in O_{m}$ and random target node $v^{\prime }$ repeatedly until we find a feasible solution.

In the inner loop, if the objective value of a neighborhood solution is less than that of a current solution, the iteration continues with the neighborhood solution as TERH is moving towards a better solution (i.e., lines 16–22). Otherwise, TERH randomly accepts the neighborhood solution with a probability in order to overcome local optimization (i.e., lines 24–28). The acceptance probability decreases with *T* for a given value of Δ. Hence, the uphill movement is more uncommon in a successive inner loop. After ϕ iterations, the inner loop finishes its solution search. After the temperature is decreased, the inner loop is started again. The approximation of TERH’s solution can be controlled by adjusting the number of iterations ϕ and cooling function C(T).

### 5.2. Reinforcement Learning Based Approximation Algorithm

We propose a Soft Actor-Critic (SAC) based approximation algorithm, called TERA, to solve the TER problem in a large-scale VCAV system. SAC is a variant of actor-critic methods for reinforcement learning. It aims to maximize expected rewards and entropy in a large-scale continuous action space [26]. While earning as many rewards as possible, it attempts to take actions as randomly as possible. This encourages the search process to discover the environment, which accelerates training and decreases the probability of going back to a visited action.

The mathematical formulation of SAC is a Markov decision process with a set of parameters including the state space M, action space A, probability density *p* and reward function *r*. The probability density *p*, defined by $M\times M\times A\to \left(0,\infty \right]$, is the probability of the next state $m_{t+1}\in M$ given the current state $m_{t}\in M$ and action $a_{t}\in A$. The reward *r*, defined by $M\times A\to \left[r_{\min},r_{\max}\right]$ is an environment reward of a state transition. SAC seeks a policy $\omega (m_{t}|a_{t}$) for maximizing the learning objective. The learning objective is the expected sum of rewards and the policy’s entropy. SAC uses the hyperparameter λ, namely temperature, to adjust the association between the reward and the entropy in the learning objective. Let *h* be the entropy function with regard to the policy ω. The formulation of the learning objective of SAC is as follows:

(27) $$\begin{array}{c} J(\omega )=\sum_{t}E_{\left(m_{t},a_{t}\right)}\left[r(m_{t},a_{t})+h(\omega (\cdot |m_{t}))\lambda \right] \end{array}$$

The primary step of developing a solution based on SAC is to formulate the three key parameters: The state space, action space, and reward function. In TERA, the state space should represent how a set of demands is satisfied when a failure happens. Hence, we formulate it by a list of tuples, $m_{t}=\left\{\left(d,u,v\right)\right\}\cup \left\{\lambda_{v}^{n}\right\}\cup \left\{\lambda_{v}^{l}\right\}$. An element of $m_{t}$ show that node *v* provides VNF *u* of service demand *d* in a failure scenario. An action in TERA makes a movement between states, representing a possible resource allocation solution. We represent an action by $a_{t}=\left(v_{1},v_{2},\ldots ,v_{|m_{t}|}\right)$ where $v_{i}\in V$ is a resource allocation solution for the *i*th tuple in the action space. As TERA optimizes the learning policy to maximize the learning objective, we use the objective function $U_{a}=-U$ to compute the reward of a solution. Hence, we can evaluate the solution’s cost efficiency and learning policy produced by TERA for minimizing the service disruption cost.

We present the main steps of TERA in Algorithm 2. The actor network returns a resource allocation action according to an input state. The NFV environment runs the action to move to a new state. The critic network uses the new state, its reward and the previous state to compute the advantage of the new state, which is used to update the weights of the actor and critic networks. The role of the critic network is the actor’s loss function. We implement the actor and critic networks as neural networks. We will discuss some details of selecting their parameters in Section 6.1.
**Algorithm 2** Learning-based approximation algorithm for TER1:**function** TERA(*G*, Γ, $y_{1}$)2:    actor← Initialize the actor network3:    critic← Initialize the critic network4:    env← Initialize the VCAV environment5:    **for** $i=1,2,\ldots ,\phi_{a}$ **do**6:        $a_{t}=\left(v_{1},v_{2},\ldots ,v_{|m_{t}|}\right)\leftarrow actor\left(m_{t}\right)$7:        $m_{t+1}=\left\{\left(d,u,v\right)\right\}\cup \left\{\lambda_{v}^{n}\right\}\cup \left\{\lambda_{v}^{l}\right\},r_{t+1}\leftarrow env\left(a_{t},m_{t}\right)$8:        $\delta \leftarrow critic(m_{t},m_{t+1},r_{t+1})$9:        Use δ to update the actor and critic networks10:    **end for**11:    **return** actor12:**end function**

## 6. Evaluation

We evaluate the service disruption cost and computation time of our proposed solution approaches for traffic engineering in a failure scenario of a VCAV system. We used the optimal solution obtained by TERO as a baseline solution for evaluating the approximation solution achieved by TERH and TERA.

### 6.1. Scenarios and Parameters Setting

Our objective is to evaluate the performance of TERO, TERH and TERA with respect to the service disruption cost and computation time when we consider various network topologies. The three main evaluation questions are as follows: What is the gap between the optimal results and approximation solutions? How do different solution approaches respond to the dynamics of failure scenarios? Can TERH and TERA efficiently provide a VNF placement and routing solution in a large-scale scenario when a failure occurs? We use eight topologies in our evaluation. Note that it is the diversity and size of topologies that affect the answer to our questions rather than a specific topology. The first topology, referred to as Abilene, is the US backbone network composed of 12 nodes and 15 links, described in the Abilene dataset [27]. The second topology, namely Geant, is the Europe backbone network of 22 nodes and 36 links, presented in the Geant dataset [28]. The other topologies are synthetic topologies based on random graph generation algorithms, including the Barabási-Albert (BA), Waxman (WA), Erdős-Rényi (ER) models [29]. We create a small topology composed of 50 nodes and a large topology composed of 200 nodes for each random graph generation algorithm. The random graph generation tool is FNSS [30]. A BA topology is created with four nodes at first. A new node is added by connecting to four preceding nodes. The link density probability used to create a WA topology is 0.9. The edge generation probability used to create an ER topology is 0.2. We denote the small and large BA topologies by BA1 and BA2, the small and large WA topologies by WA1 and WA2, and the small and large ER topologies by ER1 and ER2. In a failure scenario, we randomly generate one node and link failure in a network topology.

We randomly create 15 demands in the Abilene and Geant topologies and 100 service demands in the BA, WA, ER topologies. The arrival and departure nodes of a service demand are randomly selected. The SFC delay is varied between one and thirty milliseconds. The range of the bandwidth demand is between 1 Gbps and 5 Gbps. We consider four types of VNFs. The number of CPU cores demanded by a VNF type for one volume of traffic is varied between one and two cores. The SFC of a service demand is randomly selected in four VNF types. We assign a bandwidth value of 80 Gbps to the capacity of all links. The edge and cloud nodes are randomly selected. The cloud node capacity is 200 cores. The edge node capacity is 50 cores. At a node, the processing delay of a VNF and the routing delay for a traffic unit is randomly generated between 10 and 100 microseconds. The value of link weight is varied between 1 and 3.

We now look at how to choose hyperparameters for the implementation of our proposed algorithms. In TERA, the temperature hyperparameter is automatically configured as described in [31]. We chose two layers for the actor and critic networks because we did not obtain a better policy when the number of layers increases beyond two. After running TERA with a varying number of neurons, we chose 32 neurons for each layer of the actor and critic networks because the policy did not significantly improve when we used a bigger value.

In TERH, the value of the end temperature is 0.1. For each temperature, the number of neighbor selections is ϕ=100. For comparison purposes, we select the parameter τ of the cooling function so that the iteration number of TERH and that of TERA is similar. The parameter τ is computed as follows:

(28) $$\begin{array}{c} \tau ={\left(\frac{T_{n}}{T_{0}}\right)}^{\frac{1}{\phi_{a}}}, \end{array}$$

where $\phi_{a}=8000$ since TERA can obtain a steady policy after eight thousand iterations.

We used an x86 computer in our evaluation. Its hardware configuration is a four-core 2.60 GHz Intel processor with 8 GB memory and an NVIDIA GeForce GTX 850M card. We solved TERO in CPLEX [32]. We implemented TERH in Java and TERA in Python with TensorFlow [33].

### 6.2. Evaluation Results

**Figure 2:**
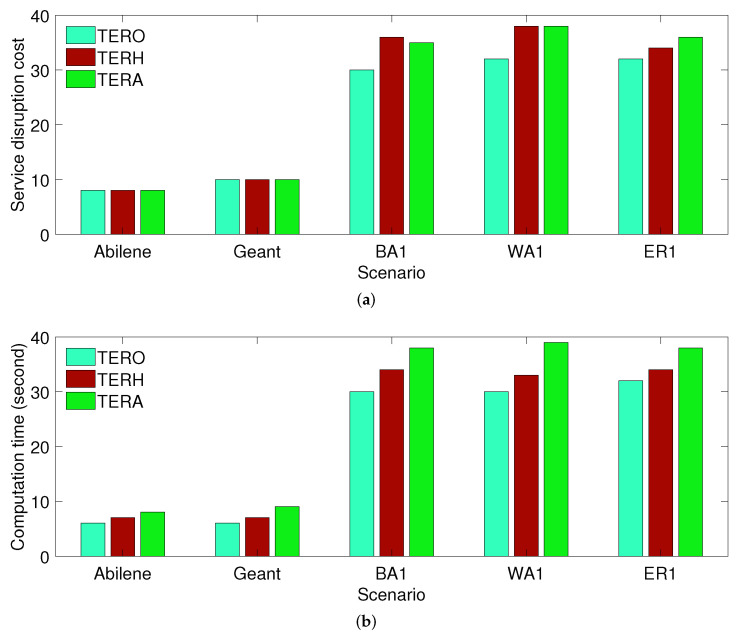
Performance comparison of TERO, TERH and TERA when a failure scenario is fixed. (**a**) Service disruption cost; (**b**) Computation time.

First, we compare the performance of different solution approaches when a failure scenario is fixed. In a fixed failure scenario, we compute the service disruption cost and computation time in only one failure scenario. We consider limited-size scenarios, including the Abilene, Geant, BA1, WA1, and ER1 topologies, to compare approximation solutions with optimal results. Figure 2a shows that TERO is better than TERH and TERA in terms of the service disruption cost, but the difference is marginal. We also observe that TERH and TERA can archive similar service disruption costs after 8000 iterations. In Figure 2b, the computation time of TERA and TERH is higher than that of TERO, and the computation time of TERA is slightly higher than that of TERH.

**Figure 3:**
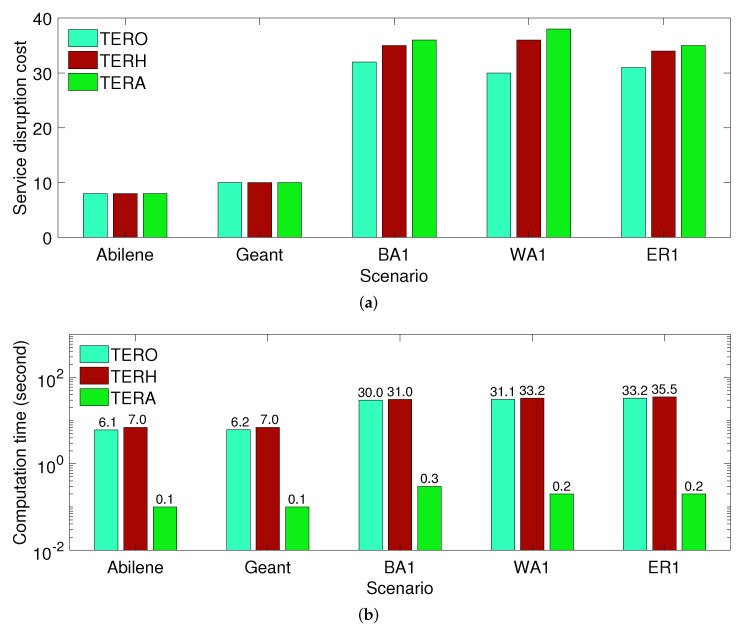
Performance comparison of TERO, TERH and TERA when a failure is changed. (**a**) Service disruption cost; (**b**) Computation time.

Second, we compare the performance of different solution approaches when a failure scenario is changed. Specifically, we consider 8000 failure scenarios in our evaluation. We compute the service disruption cost and computation time in each failure scenario and plot their average value. Figure 3a shows that TERO, TERH, and TERA archive similar service disruption costs. In Figure 3b, we use a base 10 logarithmic scale for the y-axis and a linear scale for the *x*-axis to illustrate a variation in the computation time of TERO, TERH, and TERA. The figure shows that the computation time of TERA is significantly smaller than that of TERO and TERH. It is because TERA can remember its policy learned from previous data while TERO and TERH are required to solve the TER problem for an individual failure scenario.

**Figure 4:**
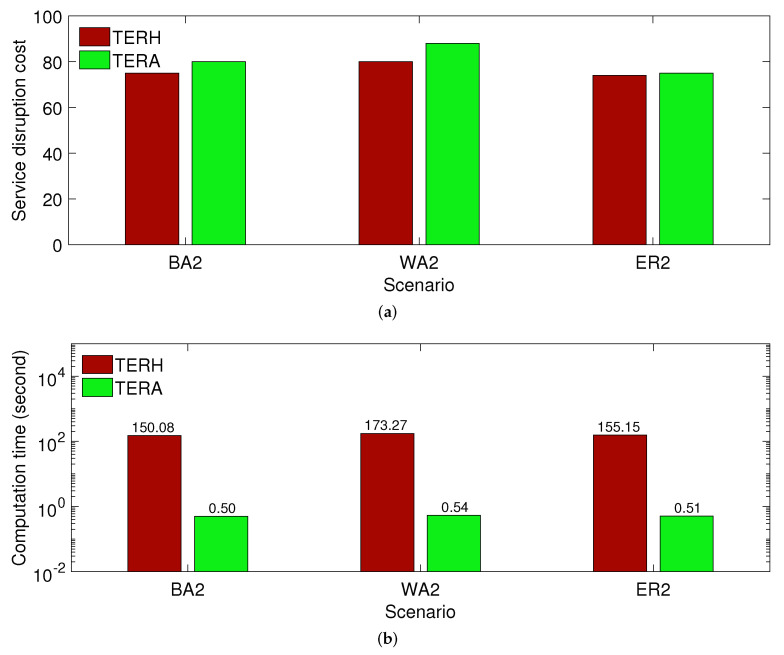
The TERA and TERH performance in a large-scale VCAV system. (**a**) Service disruption cost; (**b**) Computation time.

Finally, we evaluate the TERH and TERA performance in a large-scale VCAV system when a failure scenario is changed. Figure 4 plots the service disruption cost and computation time for the BA2, WA2, and ER2 topologies with 200 nodes. In such large-scale topologies, CPLEX cannot solve the TERO model to find the optimal solution. In Figure 4b, we use a base 10 logarithmic scale for the y-axis and a linear scale for the *x*-axis to plot the computation time. We observe that TERA is significantly faster than TERH. The service disruption cost of TERH is slightly smaller than that of TERA, but it is negligible. It suggests that we should use TERA to protect service demands from a failure in a real-time VCAV system.

## 7. Conclusions

We studied the optimization problem of traffic engineering for resilient services in a VCAV system. We proposed an ILP model (i.e., TERO) to find the optimal VNF placement and routing when a node or link failure occurs. The model captures essential features of NFV such as service function chaining, the restriction rule of resource reallocation, and the exact placement and routing solution for the service demand set. We developed the TERH and TERA approximation algorithms based on heuristics and reinforcement learning to provide an efficient traffic engineering solution for resilient services in a large-scale VCAV system. The evaluation results show that TERO, TERH, and TERA can protect service demands from node and link failures. The approximation results provided by TERH and TERA are very close to the optimal results. The results also suggest that a network service provider should consider TERA to provide resilient services in a real-time VCAV system. Possible directions for extending our work comprise the consideration of various network technologies supporting a VCAV system, an evaluation of other network topologies and performance metrics, or an optimization model of a resilient service with a federation of several VCAV providers as in [9,34].

## Figures and Tables

**Table 1:** Summary of main notations.

Input Parameters	
$G=\left(V,E\right)$	A directed graph representing a VCAV system where *V* and *E* is denoted the set of physical nodes and physical links, respectively.
$r_{v}^{n}$	Node *v*’s resource capacity
$r_{e}^{l}$	Link *e*’s bandwidth capacity
$i_{e}$	Link *e*’s beginning node
$j_{e}$	Link *e*’s ending node
*F*	The VNF type set
$\eta_{u}$	The number of CPU cores required by VNF type u∈F to process a volume of data traffic
Ω	The system-supported SFC set: $\Omega =\left\{S_{i}\right\}$, $S_{i}=\left(u_{i1},\ldots ,u_{ij},\ldots ,u_{in}\right)$; $u_{ij}$ is the *j*th VNF of SFC $S_{i}$.
$\Gamma =\left\{d\right\}$	The service demand set
$s_{d}$	Demand *d*’s arrival node
$t_{d}$	Demand *d*’s departure node
$b_{d}$	Demand *d*’s bandwidth volume
$\alpha_{d}$	Demand *d*’s SFC delay
$S_{d}$	Demand *d*’s SFC
$\beta_{v}$	The routing delay for a traffic unit at node *v*
$\mu_{vu}$	The processing delay of VNF type *u* at node *v*
$\gamma_{vv^{\prime }u_{di}}$	The moving cost when moving *i*th VNF of demand *d* from *v* to $v^{\prime }$
$\rho_{vv^{\prime }}$	The cost of the minimum-weight path between *v* and $v^{\prime }$
$\kappa_{u}$	The size of the state and data of a VNF type *u*
$\lambda_{v}^{n}$	The failure state of node *v*
$\lambda_{e}^{l}$	The failure state of link *e*
$y_{1}=\left(y_{1vdi}\right)$	The current VNF placement solution: If node *v* provides the *i*th VNF of SFC $S_{d}$, $y_{1vdi}=1$, otherwise, $y_{1vdi}=0$
$x_{1}=\left(x_{1ed}\right)$	The current routing solution: If demand *d* uses link *e*, $x_{1ed}=1$, otherwise, $x_{1ed}=0$
$w=\left(w_{e}\right)$	The weight vector of NFVI where $w_{e}$ is link *e*’s weight.
**Output variables**	
$x_{2}=\left(x_{2ed}\right)$	The routing solution for satisfying demands in the failure state: If demand *d* uses link *e*, $x_{2ed}=1$, otherwise, $x_{2ed}=0$
$y_{2}=\left(y_{2vdi}\right)$	The VNF placement solution in the failure state: If node *v* provides the *i*th VNF of SFC $S_{d}$, $y_{2vdi}=1$, otherwise, $y_{2vdi}=0$
**Auxiliary variables**	
$l_{v_{1}v_{2}}$	The length of the path from node $v_{1}$ to node $v_{2}$
θ	A large number
$y_{2vdi}^{\sigma }$	If a node between $s_{d}$ and *v* on the path realizing demand *d* provides the *i*th VNF of demand *d* (i.e., $u_{di}$), $y_{2vdi}^{\sigma }=1$, otherwise $y_{2vdi}^{\sigma }=0$.
${\bar{y}}_{2edi}^{\sigma }$	If link *e* is on the path realizing demand *d*, and a node between $s_{d}$ and $i_{e}$ on the path provides $u_{di}$, ${\bar{y}}_{2edi}^{\sigma }=1$, otherwise ${\bar{y}}_{2edi}^{\sigma }=0$.
$\lambda_{e}^{\prime }$	$\lambda_{e}^{\prime }=1$ if and only if a failure occurs on link *e*, at node $i_{e}$, or at node $j_{e}$.
$\varphi_{ed}$	$\varphi_{ed}=0$ if and only if $\lambda_{e}^{\prime }=1$ and link *e* is on the path realizing demand *d*.
$\varphi_{d}^{\sigma }$	$\varphi_{d}^{\sigma }=0$ if and only if there exists a link or node failure on the path used by demand *d*.
$z_{vv^{\prime }di}$	$z_{vv^{\prime }di}=1$ if and only if the *i*th VNF of SFC $S_{d}$ is moved from node *v* to node $v^{\prime }$, otherwise, $z_{vv^{\prime }di}=0$.
